# Influence of Deceased Donor and Pretransplant Recipient Parameters on Early Overall Kidney Graft-Survival in Germany

**DOI:** 10.1155/2015/307230

**Published:** 2015-10-11

**Authors:** Carl-Ludwig Fischer-Fröhlich, Marcus Kutschmann, Johanna Feindt, Irene Schmidtmann, Günter Kirste, Nils R. Frühauf, Ulrike Wirges, Axel Rahmel, Christina Schleicher

**Affiliations:** ^1^Deutsche Stiftung Organtransplantation, Region Baden-Württemberg, Kriegerstraße 6, 70192 Stuttgart, Germany; ^2^BQS Institute for Quality and Patient Safety, Kanzlerstraße 4, 40472 Düsseldorf, Germany; ^3^MVZ Anaesthesio Nordrhein, Hans-Günther-Sohl-Straße 6-10, 40235 Düsseldorf, Germany; ^4^Institut für Medizinische Biometrie, Epidemiologie und Informatik (IMBEI), Universitätsmedizin der Johannes Gutenberg-Universität Mainz, Obere Zahlbacher Straße 69, 55131 Mainz, Germany; ^5^Medizinische Fakultät, Albert Ludwigs Universität Freiburg, Hebelstraße 29, 79104 Freiburg, Germany; ^6^Landesärztekammer Niedersachsen, Berliner Allee 20, 30175 Hannover, Germany; ^7^Deutsche Stiftung Organtransplantation, Region Nordrhein-Westfalen, Lindenallee 29-41, 45127 Essen, Germany; ^8^Deutsche Stiftung Organtransplantation, Deutschherrnufer 52, 60594 Frankfurt am Main, Germany

## Abstract

*Background*. Scarcity of grafts for kidney transplantation (KTX) caused an increased consideration of deceased donors with substantial risk factors. There is no agreement on which ones are detrimental for overall graft-survival. Therefore, we investigated in a nationwide multicentre study the impact of donor and recipient related risks known before KTX on graft-survival based on the original data used for allocation and graft acceptance. *Methods*. A nationwide deidentified multicenter study-database was created of data concerning kidneys donated and transplanted in Germany between 2006 and 2008 as provided by the national organ procurement organization (Deutsche Stiftung Organtransplantation) and BQS Institute. Multiple Cox regression (significance level 5%, hazard ratio [95% CI]) was conducted (*n* = 4411, isolated KTX). *Results*. Risk factors associated with graft-survival were donor age (1.020 [1.013–1.027] per year), donor size (0.985 [0.977–0.993] per cm), donor's creatinine at admission (1.002 [1.001–1.004] per *µ*mol/L), donor treatment with catecholamine (0.757 [0.635–0.901]), and reduced graft-quality at procurement (1.549 [1.217–1.973]), as well as recipient age (1.012 [1.003–1.021] per year), actual panel reactive antibodies (1.007 [1.002–1.011] per percent), retransplantation (1.850 [1.484–2.306]), recipient's cardiovascular comorbidity (1.436 [1.212–1.701]), and use of IL2-receptor antibodies for induction (0.741 [0.619–0.887]). *Conclusion*. Some donor characteristics persist to impact graft-survival (e.g., age) while the effect of others could be mitigated by elaborate donor-recipient match and care.

## 1. Introduction

Donor and recipient parameters interact and jointly influence overall graft-survival after kidney transplantation (KTX). The individual decision to use a graft or not is guided by the question whether it will be helpful for the allocated recipient taking into account persisting organ shortage. As a result, grafts from elderly donors or from donors with comorbidities are used increasingly. However there is no agreement which donor factors may or may not directly affect transplant outcome in correlation to recipient factors [1]. German data beyond the scope of single-center experience were not available until now. Thereby recent studies [2–4] concluded that many grafts assumed to be marginal can be used for KTX with appropriate graft-survival. The authors requested that their results should be confirmed through analysis of the national data by a currently not existing scientific registry [2, 4]. With regard to the German donor and recipient population factors which determine the outcome of KTX should be analysed as recently done in heart and liver transplantation [5–8]. Characteristics of donors and recipients as well as graft-morphology as present right before KTX should be investigated about their impact on graft-survival to assure the safety and quality of donor and graft selection nationally by means of univariate analyses and multiple Cox regression.

## 2. Material and Methods

The present study uses data from two institutional databases. For quality assurance and patient safety reasons, data on transplantation as well as follow-up surveys were reported to the BQS Institute for Quality and Patient Safety from 2006 to 2008. In 2006 the German national organ procurement organization Deutsche Stiftung Organtransplantation (DSO) implemented a nationwide database for all donor-derived data collected prospectively and directly in the donor hospitals. These data on donor characterization were used for the allocation of kidney grafts via Eurotransplant. Merging the two databases of DSO and BQS into a completely deidentified research database allowed us to analyse the impact of donor characteristics on graft-survival after KTX. Further methodological details have been published elsewhere [5–8].

The study was performed in accordance with the guidelines for Good Scientific and Good Epidemiological Practice of the German Society for Epidemiology (DGEPI 2008) [9]. Ethical approval was not needed as we fulfilled the criteria of “Good Practice in Secondary Data Analysis.”

The study population consists of 4411 deidentified records of kidney grafts donated from brain dead donors in Germany (DBD) and transplanted as isolated grafts in different German centers between 2006 and 2008. The study was restricted to the evaluation of graft-survival because in a worst case assumption both graft-failure and returning to dialysis or death of the recipient with functioning graft can be related to factors known before the event of KTX.

In a first step, the impact of single relevant donor and recipient risk factors on graft-survival was analysed by means of log-rank tests (concerning nominal and categorical factors) and univariate Cox regression (concerning interval-scaled factors). In a second step, a multiple Cox regression model was developed to examine the joined impact of several risk factors on graft-survival. In this model, only risk factors which showed a *P* value below 0.20 in univariate analysis and existed before the event of KTX were considered initially. A *P* value below 0.05 in multivariate analysis was considered as significant (stepwise forward selection). All analyses were performed with IBM SPSS Statistics 19 (SPSS Inc., Chicago, IL, USA).

For donor laboratory data after admission first, last, lowest, and peak value before procurement were analysed.

Graft-quality was judged as good, medium, or poor according to the assessment of the surgeon at procurement based on visual inspection [10]. This is common practice and formally agreed upon within the Eurotransplant (ET) countries (including Austria, Belgium, Croatia, Hungary, Luxemburg, Slovenia, Netherlands, and Germany) for decades. Nevertheless beyond this subjective grading any relevant anatomical or pathological finding must be described in detail [10].

Percentages of peak and actual panel reactive antibodies (PRA) were calculated after screening with the complement dependent lymphocytotoxicity test [11]. Unfortunately precise data about HLA-A, HLA-B, and HLA-DR mismatches were not available for most recipients of grafts of donors >64 years of age (*n* = 1364) due to allocation rules. Therefore this could not be considered in analysis.

Graft-survival-times were calculated from the data on postoperative hospital stay and follow-up examination. Mean graft-survival-time was 120.7 days for recipients whose kidney graft failed during the study period and 358.6 days for censored cases. In case of a recipient follow-up missing graft-survival was censored for the most recent data actually available. Due to legal rules follow-up had to be terminated at 3 years.

## 3. Results

4411 kidneys were donated and transplanted in Germany between 2006 and 2008. 2085 were recovered from female and 2326 from male donors. 1634 kidneys were transplanted into female recipients and 2777 into male recipients. The gender match does not reveal any differences in graft-survival. Further details about donor and recipient characteristics are summarized in Tables 1 and 2 and in Supplemental Tables e1 and e2 (see Supplementary Material available online at http://dx.doi.org/10.1155/2015/307230).

### 3.1. Univariate Analysis

#### 3.1.1. Basic Donor Characteristics and Donor History

Univariate analyses showed that graft-survival is significantly influenced by increasing donor age, limited donor size, atraumatic cause of donor death, preexisting arterial hypertension, and coronary heart disease or reactive antibody status against cytomegaly virus (Table 1).

Graft-survival is not compromised significantly by other donor characteristics (Table 1) as well as most laboratory parameters documented according to the rules [10] (Table e1). Some insignificant results are remarkable (e.g., smoking history or acute events of cardiac resuscitation).

#### 3.1.2. Donor Management

Some medications used during donor maintenance are of significant protective effect on graft-survival (Tables 1 and e1) such as the application of any kind of catecholamines at time of report to Eurotransplant or within 24 hours before, for example, norepinephrine.

#### 3.1.3. Procurement, Allocation Issues, and Ischemia Time

Graft and preservation quality (assessed as good, medium, or poor by the surgeons at procurement) is of significant impact on graft-survival. Parameters describing kidney anatomy prospectively at procurement such as side of the kidney (left or right), the number of arteries or veins, and length of the ureter and kind of preservation solution used are without significant influence on graft-survival as well as rescue allocation or local versus national exchange.

**Figure 1:**
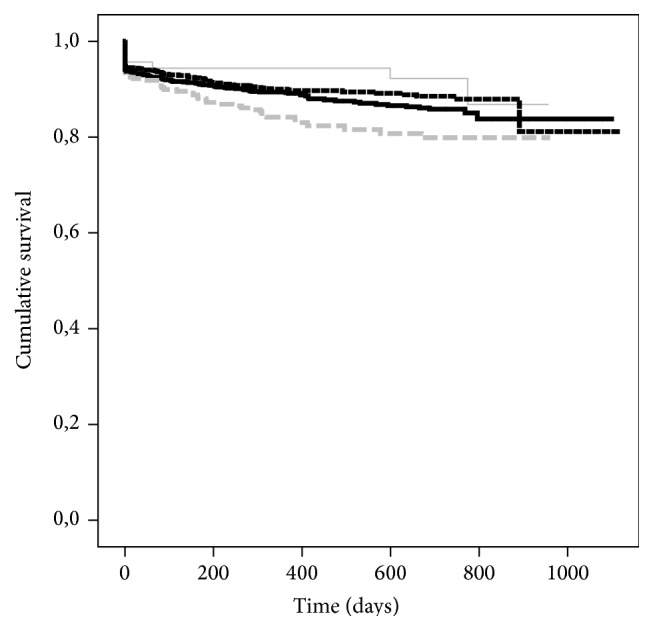
Kaplan-Maier estimates of graft-survival for risk factor cold ischemia time for all cases with donor age <65 years: grouping for intervals of ischemia times for 0–12 h (*n* = 1195, black dashed solid line), 12–18 h (*n* = 1323, black solid line), 18–24 h (*n* = 413, grey dashed line), and >24 h (*n* = 116, grey solid line); *P* = 0.010.

**Figure 2:**
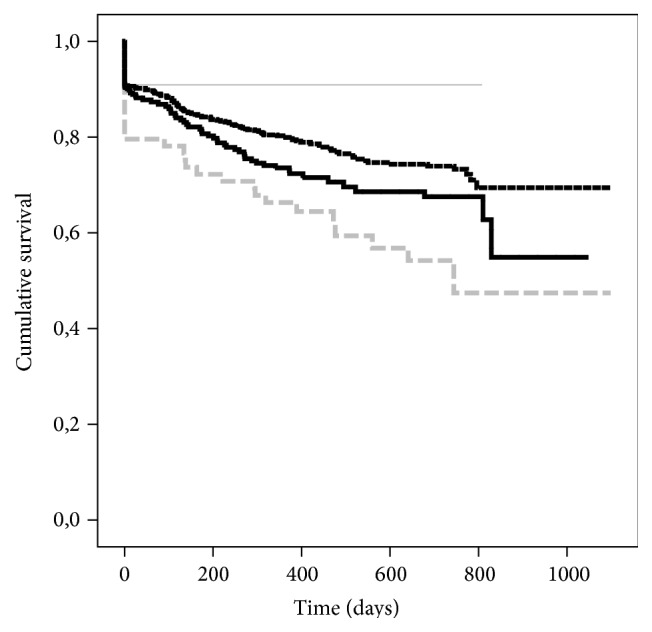
Kaplan-Maier estimates of graft-survival for risk factor cold ischemia time for all cases with donor age ≥65 years: grouping for intervals of ischemia times for 0–12 h (*n* = 897, black dashed line), 12–18 h (*n* = 352, black solid line), 18–24 h (*n* = 93, grey dashed line), and >24 h (*n* = 22; grey solid line); *P* = 0.002.

Cold ischemia time (CIT) has a pivotal role for graft-survival, particularly in the group of donors above 65 years of age (Figures 1-2). We analysed those grafts separately because the Eurotransplant Senior Program (ESP) [10, 11] aims to decrease CIT by allocating them regionally while omitting delays through HLA-typing. Moreover, these grafts are allocated exclusively to recipients older than 65 years. CIT > 12 h is a risk factor in this donor age group (Figure 2). The apparently better graft-survival of grafts with CIT of more than 24 is negligible because of the low number of cases and the exception of high quality grafts.

#### 3.1.4. Recipient Characteristics

**Figure 3:**
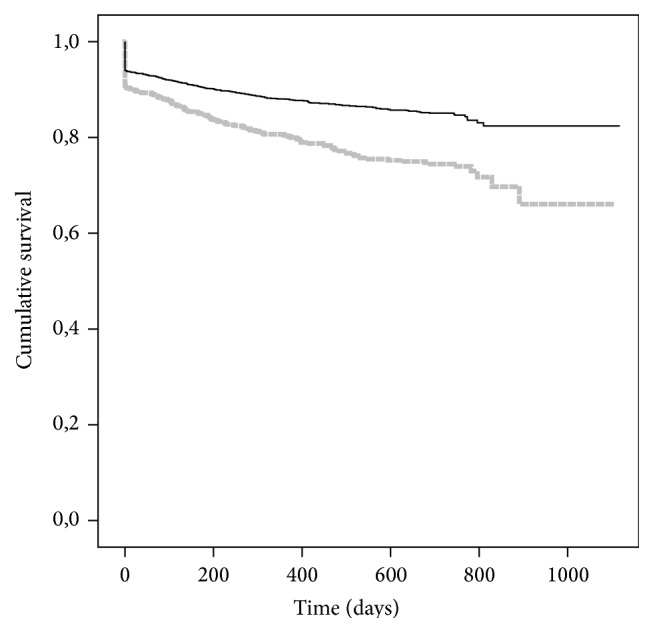
Kaplan-Maier estimated for graft-survival after kidney transplantation (KTX) according to preexisting cardiovascular morbidity of the recipient including diabetes: none (*n* = 2932; black line), yes (*n* = 1479; grey line); *P* < 0.001.

Recipient age is of significant impact on graft-survival (Tables 2 and e2). Furthermore, cardiovascular comorbidities like diabetes, coronary heart disease, or peripheral vascular artery disease limit graft-survival significantly (Figure 3). After an average time of 5.8 ± 3.3 years from start of dialysis until KTX no impact of this time on graft-survival exists.

**Figure 4:**
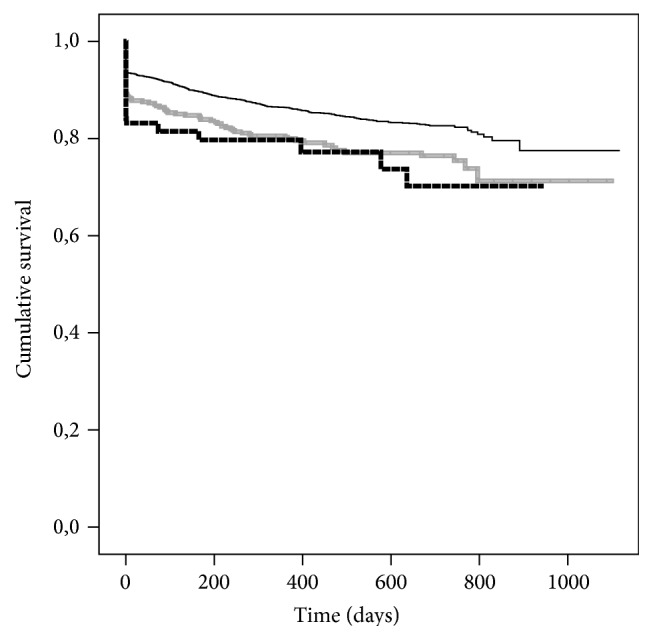
Kaplan-Maier estimated for graft-survival after kidney transplantation (KTX) according to number of previous KTX: none (*n* = 3779; black solid line), one (*n* = 525; grey solid line), and more than one (*n* = 107; dashed black line); *P* ≤ 0.001.

**Figure 5:**
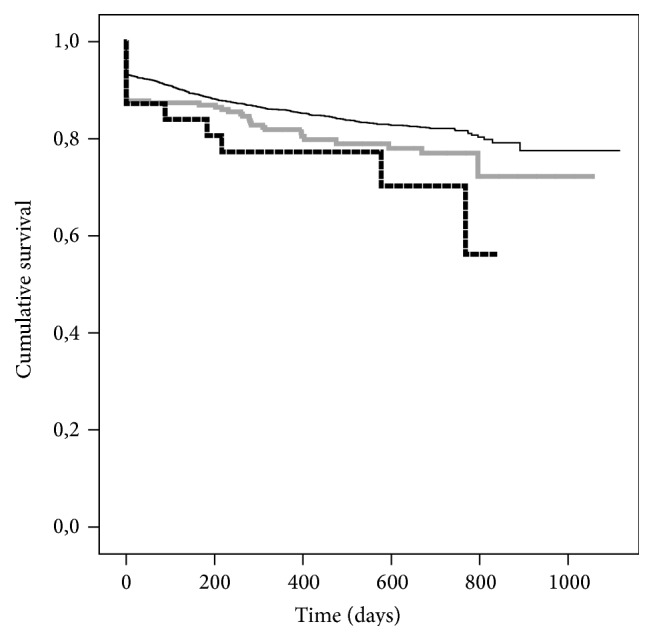
Kaplan-Maier estimated for graft-survival after kidney transplantation (KTX) according to actual degree of panel reactive antibodies against HLA-antigens: 0–5% (*n* = 4019; black solid line), 6–84% (*n* = 345; grey solid line), and 85–100% (*n* = 47; dashed black line); *P* = 0.007.

If patients need re-KTX, the risk of graft loss increases significantly. This seems to be linked to the current degree of immunization against HLA-antigens as the Kaplan-Maier survival-plots are very similar in their trend (Figures 4-5), mainly due to early failures within postoperative period.

Beyond duration of transplantation surgery, postoperative hospital stay and need for postoperative dialysis postoperative complications are another significant risk factor concerning graft-survival. In the study population three levels existed according to their impact on graft-survival (Table 2):

(1) marginal effect on graft-survival (*n* = 330): urine leakage, lymphocele,
(2) compromising effect on graft-survival (*n* = 386): wound dehiscence, severe bleeding, wound infection, and other,
(3) disastrous effect on graft-survival (*n* = 65): arterial or venous thrombosis.

### 3.2. Multiple Analyses

Multiple Cox regression of factors known before the event of KTX shows (Table 3) that graft-survival is influenced by donor age, medium or poor organ quality assessed at procurement, and increased creatinine values of the donor at admission. Protective effects can be assumed for catecholamine treatment during donor maintenance and donor size. Significant recipient variables are age, preexisting cardiovascular diseases, actual PRA, retransplantation, and the type of induction therapy used at KTX. The not significant benefit of IL2-receptor antibody treatment observed in univariate analysis became a significant protective marker if considered simultaneously with other factors in multiple analyses. This statement is limited by the fact that we lack information concerning specific indications for induction therapy (e.g., interaction of immunization and recipient age). All other organ, donor, and recipient characteristics significant in univariate analysis are not significant in the multiple model.

## 4. Discussion

This is the first national investigation for Germany which takes into account joint donor and recipient factors on a multicenter-level known before the event of KTX. In contrast to other studies, the analysed donor data are real-time data used for organ allocation and terminal decisions by recipient centers to realize KTX. The corresponding recipient data were collected for quality assurance reasons according to German law. To ensure data consistency, our study was limited to KTX performed in Germany with grafts recovered in Germany only.

Coordinators and procurement-teams are familiar with the risks factors identified by donor characterization, donor evaluation, and graft assessment at procurement [12]. According to the recommended risk-benefit-assessment, recipient centers take into account further the actual health status of the recipient (e.g., cardiovascular comorbidity, immunisation events) when a graft is accepted for a particular recipient [12–15]. Therefore, our analyses may be affected by this selection bias caused by the process of donation and allocation.

In multiple Cox regression analysis (Table 3) increased donor age and assessed reduced graft-quality at procurement are associated with lower graft-survival. As expected this is in accordance with other studies [16–23]. However, according to our multiple model graft-survival is not limited significantly by cardiovascular risks of the donor like arterial hypertension, diabetes, cause of death due to cerebrovascular accident, or smoking. This is probably due to the fact that the influence of these risks is already covered by increased age in our population. Another cofounder can be the assessment of graft-quality as reduced (medium or poor) by the procurement surgeon. But this is always subjective and therefore associated with limitations in validity [1] because such grafts are discarded by some centres whereas others transplant them with success [2–4, 24–27]. The authors expect that by improved quality of the donation-allocation-transplantation process risk factors like graft-quality, donor maintenance, or laboratory parameters may become negligible.

The use of vasopressors or catecholamines does not compromise graft-survival, which underpins the findings of Schnuelle et al. [28]. Thereby no negative dosing effect of norepinephrine seems to exist. Actual vasopressor support mandatory for compensating vasoplegia after brain death appears to be harmless as long as appropriate volume therapy had been initiated before. The protective effect of donor size can be attributed to the fact that undersized donor-grafts may not provide enough nephron mass for the recipient.

No appropriate evidence exists on which renal function parameters should be used for the assessment of kidney function. In this population only in multiple analyses creatinine values measured initially at admission are significantly related to graft-survival. This must be interpreted with caution since potential donors are not in a stable status as it is required for assessment of kidney function directly or indirectly by creatinine measurement [29] with nonlinear relation to function. How to identify acute on chronic damage will be challenging since acute kidney injury (AKI) itself is probably not limiting graft-survival [2]. Despite volume resuscitation within appropriate intensive care therapy and care of diabetes insipidus [13, 30] an overlay of reversible AKI after renal hypoperfusion must be considered due to primary devastating cerebral complications.

The issue of donor age and ischemia time (CIT) deserves careful attention because in the whole population in 84.7% of the cases CIT did not exceed 18 hours. When using the limit of CIT below 12 hours as reference and analysing only donors younger than 65 years of age no impact on graft-survival was observed for cases with CIT between 12 and 18 hours while graft-survival was significantly decreased if CIT exceeded 18 hours (Figure 1). But when analysing the only donors older than 65 years of age, increasing CIT above 12 hours already compromised graft-survival significantly (Figure 2) in univariate analysis. Therefore in accordance with other studies and guidelines [1, 14, 16, 17, 23, 31] it must be recommended to maintain CIT as short as possible, since advanced donor age and prolonged CIT are a detrimental combination. There were a few cases in population where acceptable results had been achieved despite prolonged CIT. This must be attributed to a selection of special cases in combination of recipient issues and graft-quality despite donor age, where long CIT is a calculated risk compared to the risk of not transplanting the recipient and discarding the graft. Since CIT is within a narrow time frame in this population and donor age is already a significant risk factor in multiple Cox regression it can be expected that CIT is not included in the model due to the outlined relation.

Donor related factors such as those summarizing disease transmission risks [32, 33], describing anatomical variants of a graft prospectively, events of cardiac resuscitation or hypotension (for definition see [10]), duration of hospital stay, monitoring values, body mass index, and numerous laboratory parameters (as requested [10]) have no significant impact on graft-survival.

The impact of CIT and other factors on delayed graft function (DGF) was not investigated further because DGF occurs after the event KTX. Therefore concerning graft-survival DGF is a risk connected through by the risks known before KTX and created in addition afterwards.

Concerning recipient related risk factors, multiple Cox regression analysis (Table 3) reveals that increased age, cardiovascular disease including diabetes, and immunization events in the HLA system (expressed as panel reactive antibodies and/or retransplantation) have a negative effect on graft-survival. This confirms that careful recipient evaluation and selection are crucial for good transplantation results [14].

Some preparations used for induction therapy are without significant impact on graft-survival (e.g., ATG) while others turned out to be beneficial (e.g., IL2-receptor antibodies) in our analysis. This underpins the guidelines [14, 15]. However, the interpretation of this result is difficult since other immunosuppressive drugs were used and combined heterogeneously (data not shown) and immunosuppressive protocols are based on the discretion of the recipient centers. Evaluation of immunosuppressive protocols was not the scope of this study.

Patients with chronic renal disease (CKD) suffer from comorbidities. While a previous history of arterial hypertension is without significant impact on graft-survival, as expected, recipients with diabetes, coronary heart disease, and/or peripheral vascular artery disease are at risk for decreased graft-survival. We could not detect a significant relation between graft-survival and the time spent on dialysis before KTX at an average time of 5.8 ± 3.3 years in this study while patients on the waiting list are managed according to the guidelines [14, 15].

In this study posttransplantation variables are not considered in multiple analysis because we investigated the interaction of donor and recipient parameter known until the moment of decision making whether an allocated organ will be transplanted on the selected recipient or not. However, the data reported to the BQS Institute for reasons of quality assurance include data concerning the postoperative course which should be investigated separately. According to the national guidelines [32, 33] rescue allocation took place in 5.5% of the KTX and 79.2% of all grafts are exchanged between procurement centers and recipient centers without significant impact on graft-survival. Therefore, allocation rules seem to be safe.

For donors below 65 years of age zero HLA-B or zero HLA-DR broad antigen mismatches seem to be beneficial concerning graft-survival. Due to technical restrictions no data about HLA-mismatches became available for donors within ESP, which caused an inacceptable rate of missing values for further multivariable analysis.

Intensive care units in donor hospitals are liable for their treatment protocols which are in compliance with national and international recommendations [13, 30, 34]. Some medications vary in contrast to other countries (e.g., desmopressin is used as an antidiuretic hormone; norepinephrine is preferred as vasopressor). Desmopressin is merely marginal protective on graft-survival, which partially confirms the results of Benck et al. [35]. When diuretics are used during donor care then probably compromising obstacles have occurred before which compromise graft-survival. On the contrary the use of colloids in fluid management shows no impact on graft-survival (Table e1) although we did not differentiate between the different kinds of colloids. The question if hydroxyethyl starch explicitly impacts graft-survival became irrelevant since it should not be used anymore for critically ill patients in Germany [36].

A limitation of the study is the short follow-up period, but when implementing the concept of mandatory quality assurance in medicine prospectively by law it was decided to follow up recipients only for three years. In the future, such programs should include longer follow-up periods within a transplant registry as requested by others [2, 4]. However, this study contributes important knowledge on how to merge multiple institutional databases without conflict of interests and with protection of patient rights. This knowledge gained can be used to establish an effective transplantation registry. At least for factors with impact on early graft-survival conclusion can be drawn. Whether to exclude KTX combined with other organs or not can be discussed. However, for an initial evaluation of the joint impact of donor and recipient parameters it was helpful to exclude such factors.

Preimplantation or zero-time biopsy data were not available since there is no national consensus on how to perform, standardize, and merge this information including the pragmatic recommendation to do this according to the Banff classification in line with all other transplant biopsies taken later on [37, 38]. Preimplantation biopsy is not done systematically in all ET countries including Germany at this moment due to the fact that there is general agreement in the ET community that the added value of routine kidney biopsies for evaluating donor kidney quality is limited if it comes to predicting intermediate and long-term function of the donated kidneys. Therefore kidney biopsies are currently performed only for specific indications, for example, exclusion of suspected tumor at time of procurement. These limited data are systematically collected and stored in the original donor database.

Since this was the first national study using all recipient and donor data as they existed right before KTX we did not include expanded donor criteria definitions or donor risk index calculations [21–23, 39]. They are all derived from other donor-recipient populations. But out of the multiple donor characteristics used to calculate them in our multiple Cox regression analysis only donor age and size are of significant impact on graft-survival while in our population use of vasopressors (protective) and subjective assessment of graft-quality at procurement are significant risk factors. Therefore without continuous further validation and adjustment to the national population such definitions should not be used especially when their predictive value lacks appropriate evidence according to the review of Dare et al. [1].

## 5. Conclusion

Beyond the crucial functional and morphological assessment of a graft before KTX only donor age limits graft-survival significantly as unchangeable risk factor besides graft assessment at procurement and estimation of irreversible impaired renal function of the donor. On the recipient side age, cardiovascular comorbidity and immunological status are significant risk factors which determine graft-survival. This has to be considered in the detailed and complete characterization of the donor organ and the elaborate recipient selection when discussing further how to handle the combination of donor and recipient related risk factors when caring for recipients with different probabilities of long-term survival.

## Supplementary Material

In the supplementary all data investigated are shown. They which were used for the Cox Regression model devlopement accoridng to the details outlined in the mehtods section. Table e1 summarizes all donor data, table e2 summarizes all recipient data.

## Figures and Tables

**Table 1:** Summary of donor characteristics and transplant variables used in univariate analyses of graft-survival after isolated kidney transplantation (KTX). For interval-scaled parameters absolute numbers, median, interquartile range, risk ratio (with 95% confidence interval), and *P* values (Cox regression) are shown. For nominal and categorical parameters absolute numbers, proportions, the percentage of graft-failures, and *P* values (log-rank test) are shown. All donor characteristics and transplant variables investigated are shown in supplementary Table e1.

Donor characteristics and basic donor data	Unit of analysis or factor level	*N* at risk	(%)	Median	Interquartile range	Graft-failure (%) or hazard ratio [95% CI]	*P* value
Age	Year	4392		56	45–67	1.027 [1.021–1.033]	<0.001
Gender	Female	2085	(47.3)			14.0%	0.490
	Male	2326	(52.7)			13.5%	
Weight	kg	4411		80.0	70.0–90.0	0.998 [0.993–1.002]	0.320
Size	cm	4411		172.0	165.0–180.0	0.984 [0.977–0.992]	<0.001
Stay in intensive care unit	day	774		4.0	2.0–8.0	0.987 [0.970–1.005]	0.148
Cause of death	Cerebral hypoxia	557	(12.6)			11.7%	0.003
	CVA (bleeding)	2516	(57.0)			14.9%	
	Ischemic stroke	497	(11.3)			14.7%	
	Other	78	(1.8)			1.3%	
	Trauma	763	(17.3)			11.9%	
Cardiac resuscitation [10]	None	3876	(87.8)			14.0%	0.094
	Any	535	(12.1)			11.8%	

*Procurement and allocation*							
Time of death until cross clamp							
(i) With procurement of thoracic organs	Hour	1900	(43.1)	12.3	10.0–15.9	0.988 [0.964–1.011]	0.306
(ii) Without procurement of thoracic organs	Hour	2511	(56.9)	8.8	6.6–11.4	0.985 [0.967–1.003]	0.111
Ischemia time	Minute	4411		741.0	544.0–946.0	1.000 [1.000–1.000]	0.544
Preservation solution	HTK	3804	(86.2)			13.7%	0.217
	UW	597	(13.5)			13.9%	
	Other	10	(0.2)			30.0%	
Graft-quality at recovery	Good	3965	(90.7)			12.8%	<0.001
	Poor or medium	405	(9.3)			23.7%	

*Medication (at ET report)*							
Catecholamines (actual)	No	1138	(25.8)			16.2%	0.016
	Yes	3273	(74.2)			12.9%	
Catecholamines within last 24 hours	No	765	(17.3)			16.2%	0.035
	Yes	3646	(82.7)			13.2%	

*Additional diagnosis*							
History of arterial hypertension	Not reported	2568	(58.2)			12.3%	0.001
	Reported	1843	(41.8)			15.7%	
History of diabetes	Not reported	4240	(96.1)			13.6%	0.097
	Reported	171	(3.9)			17.5%	
History of coronary heart disease	Not reported	3584	(81.3)			13.3%	0.027
	Reported	827	(18.7)			15.7%	
History of smoking	Not reported	3177	(72.0)			14.3%	0.181
	Reported	1234	(28.0)			12.4%	
History of contact to cytomegaly virus	Anti-CMV−	1723	(39.1)			12.0%	0.022
	Anti-CMV+	2688	(60.9)			14.8%	

*Laboratory data*							
Creatinine at admission	*μ*mol/L	4400		76.9	61.9–97.2	1.001 [1.000–1.003]	0.104
Creatinine at ET report	*μ*mol/L	4399		79.6	61.9–110	1.000 [0.999–1.002]	0.389

**Table 2:** Summary of recipient characteristics used in univariate analyses of graft-survival after isolated kidney transplantation (KTX). For interval-scaled parameters absolute numbers, median, interquartile range, risk ratio (with 95% confidence interval), and *P* values (Cox regression) are shown. For nominal and categorical parameters absolute numbers, proportions, the percentage of graft-failures, and *P* values (log-rank test) are shown. All recipient characteristics investigated are shown in supplementary Table e2.

Basic recipient data	Unit of analysis or factor level	*n*	(%)	Median	Interquartile range	Graft-failure (%) or hazard ratio [95% CI]	*P* value
Age	Year	4411		56	46–65	1.028 [1.021–1.035]	<0.001
Weight	kg	4411		75.0	65.0–86.0	1.003 [1.000–1.006]	0.057
Size	cm	4411		172.0	165.0–178.0	0.996 [0.991–1.002]	0.164
Time on dialysis before KTX	Days	4346		2251	1239–2823	1.000 [1.000–1.000]	0.141
Gender	Female	1634	(37.0)			12.7%	0.145
	Male	2777	(63.0)			14.3%	

*Comorbidities before KTX*							
Diabetes	Not reported	4035	(91.5)			13.4%	<0.001
	Reported	376	(8.5)			17.0%	
Coronary heart disease	Not reported	3351	(76.0)			11.9%	<0.001
	Reported	1060	(24.0)			19.4%	
Peripheral artery occlusion disease	Not reported	3908	(88.6)			12.8%	<0.001
	Reported	503	(11.4)			21.1%	

*Immunological risks before KTX*							
Retransplantation	1st KTX	3779	(85.7)			12.7%	<0.001
	2nd KTX	525	(11.9)			19.4%	
	3rd or more KTX	107	(2.4)			21.5%	
Panel reactive antibody (peak value)	0–5%	3511	(79.6)			13.2%	0.020
	6–84%	745	(16.9)			15.2%	
	85–100%	155	(3.5)			20.0%	
Panel reactive antibody (last value before KTX)	0–5%	4019	(91.1)			13.3%	0.007
	6–84%	345	(7.8)			18.0%	
	85–100%	47	(1.1)			23.4%	

*Postoperative course of KTX*							
Postoperative stay in hospital	Days	4411		21	16–29	1.018 [1.016–1.021]	<0.001
Duration of KTX-operation	Minutes	4411		160	129–198	1.002 [1.000–1.003]	0.009
Postoperative dialysis	Number	4103		0	0–2	1.078 [1.062–1.095]	<0.001
Rejections postoperative	0	3667	(83.1)			11.9%	<0.001
	1	635	(14.4)			20.3%	
	≥2	109	(2.5)			38.5%	
Induction therapy at KTX	None	1772	(40.2)			15.0%	0.162
	ATG, OKT3, or other	668	(15.1)			15.1%	
	IL2-receptor antibodies	1971	(44.7)			12.2%	
Postoperative complications (worst case scenario in case of multiple counts)	Not reported	3630	(82.2)			11.3%	<0.001
	(1) Urine leak or lymphocele	330	(7.5)			12.4%	
	(2) Wound infection, dehiscence	386	(8.8)			25.9%	
	(3) Thrombosis (a. or v. renalis)	65	(1.5)			83.1%	

**Table 3:** Results of multiple Cox regression analysis concerning graft-survival after isolated kidney transplantation (KTX, *n* = 4411). Data analysis was preceded by a donor-recipient selection process during graft allocation. Only donor and recipient risk factors known before kidney transplantation were considered (all variables listed in Tables e1 and e2 with *P* < 0.2 were considered for selection into the model).

	Hazard ratio	95% confidence interval		*P* value
*Donor related risk factors (unit of analysis or factor level)*				
Age (years)	1.020	1.013	1.027	0.000
Size (cm)	0.985	0.977	0.993	0.001
Creatinine at admission (*µ*mol/l)	1.002	1.001	1.004	0.002
Catecholamines before recovery	0.757	0.635	0.901	0.002
Quality of graft (reduced versus good)	1.549	1.217	1.973	0.000

*Recipient related risk factors (unit of analysis or factor level)*				
Age (years)	1.012	1.003	1.021	0.006
Actual PRA (%)	1.007	1.002	1.011	0.006
Retransplantation (yes versus no)	1.850	1.484	2.306	0.000
Cardiovascular disease (yes versus no)	1.436	1.212	1.701	0.000
Induction therapy (none = references)				0.005
(i) Interleukin 2 receptor antibodies	0.741	0.619	0.887	0.001
(ii) Other antibodies (e.g., ATG, OKT3)	0.838	0.665	1.055	0.133

PRA: panel reactive antibodies; catecholamines: norepinephrine, epinephrine, dopamine, or dobutamine; cardiovascular disease: coronary heart disease or peripheral arterial occlusion disease or cerebrovascular insult or diabetes.
