# Supplementary material for: Influence of Deceased Donor and Pretransplant Recipient Parameters on Early Overall Kidney Graft-Survival in Germany
Source: J Transplant. 2015 Oct 11;2015:307230. doi: 10.1155/2015/307230 (PMC4619958; doi:10.1155/2015/307230)
Supplement: Supplementary file 1 — In the supplementary all data investigated are shown. They which were used for the Cox Regression model devlopement accoridng to the details outlined in the mehtods section. Table e1 summarizes all donor data, table e2 summarizes all recipient data. [file 307230.f1.doc]

**Supplementary Material:**

**Table e1:** Donor characteristics and transplant variables used in univariate analyses of graft-survival after isolated kidney transplantation (KTX). For interval-scaled parameters absolute numbers, median, interquartile range, risk ratio (with 95% confidence interval) and p-values (Cox regression) are shown. For nominal and categorical parameters absolute numbers, proportions, the percentage of graft-failures and p-values (log-rank test) are shown.

| **Donor characteristics and basic donor data** | | | | | | **Unit of analysis or factor level** | | | | **N at risk** | **(%)** | | **Median** | | **Interquartile range** | **Graft-failure (%) or Hazard Ratio [95%-CI]** | **p-value** |
| --- | --- | --- | --- | --- | --- | --- | --- | --- | --- | --- | --- | --- | --- | --- | --- | --- | --- |
|
| Age | | | | | | year | | | | 4392 |  | | 56 | | 45-67 | 1.027 [1.021-1.033] | <0.001 |
|  | | | | | | age<65 years | | | | 3028 | (69.0) | |  | |  | 10.4% | <0.001 |
|  | | | | | | age≥65 years | | | | 1364 | (31.0) | |  | |  | 20.9% |  |
| Gender | | | | | | female | | | | 2085 | (47.3) | |  | |  | 14.0% | 0.490 |
|  | | | | | | male | | | | 2326 | (52.7) | |  | |  | 13.5% |  |
| Weight | | | | | | kg | | | | 4411 |  | | 80.0 | | 70.0-90.0 | 0.998 [0.993-1.002] | 0.320 |
| Size | | | | | | cm | | | | 4411 |  | | 172.0 | | 165.0-180.0 | 0.984 [0.977-0.992] | <0.001 |
| Body Mass Index (BMI) | | | | | | kg/m² | | | | 4411 |  | | 25.7 | | 23.9-28.4 | 1.010 [0.994-1.027] | 0.206 |
| Stay in intensive care unit | | | | | | day | | | | 774 |  | | 4.0 | | 2.0-8.0 | 0.987 [0.970-1.005] | 0.148 |
| Cause of death | | | | | | cerebral hypoxia | | | | 557 | (12.6) | |  | |  | 11.7% | 0.003 |
|  | | | | | | CVA (bleeding) | | | | 2516 | (57.0) | |  | |  | 14.9% |  |
|  | | | | | | Ischemic stroke | | | | 497 | (11.3) | |  | |  | 14.7% |  |
|  | | | | | | other | | | | 78 | (1.8) | |  | |  | 1.3% |  |
|  | | | | | | trauma | | | | 763 | (17.3) | |  | |  | 11.9% |  |
| Heart rate | | | | | | BPM | | | | 4116 |  | | 93.0 | | 81.0-109.0 | 0.998 [0.994, 1.002] | 0.300 |
| Diuresis within last 24h | | | | | | l | | | | 3985 |  | | 4000.0 | | 2850.0-5458.5 | 1.000 [1.000-1.000] | 0.054 |
| Diuresis last hour (actual) | | | | | | l | | | | 4039 |  | | 150.0 | | 100.0-240.0 | 1.000 [0.999-1.000] | 0.356 |
| Body temperature (actual) | | | | | | °C | | | | 4072 |  | | 36.5 | | 36.0-37.1 | 0.974 [0.899-1.055] | 0.519 |
| Cardiac resuscitation [10] | | | | | | none | | | | 3876 | (87.8) | |  | |  | 14.0% | 0.094 |
|  | | | | | | any | | | | 535 | (12.1) | |  | |  | 11.8% |  |
| Hypotensive periods [10] | | | | | | none | | | | 4049 | (91.8) | |  | |  | 13.8% | 0.348 |
|  | | | | | | any | | | | 362 | (8.2) | |  | |  | 12.7% |  |
| **Procurement and allocation** | | | | | |  | | | |  |  | |  | |  |  |  |
| Time death until cross clamp | | | | | |  | | | |  |  | |  | |  |  |  |
| - with procurement of thoracic organs | | | | | | | | | hour | 1900 | (43.1) | | 12.3 | | 10.0-15.9 | 0.988 [0.964-1.011] | 0.306 |
| - without procurement of thoracic organs | | | | | | | | | hour | 2511 | (56.9) | | 8.8 | | 6.6-11.4 | 0.985 [0.967-1.003] | 0.111 |
| Ischemia time | | | | | | minute | | | | 4411 |  | | 741.0 | | 544.0-946.0 | 1.000 [1.000-1.000] | 0.544 |
|  | | | | | | ≤12 hours | | | | 2092 | (47.4) | |  | |  | 13.4% | 0.001 |
|  | | | | | | 12 - ≤18 hours | | | | 1675 | (38.0) | |  | |  | 13.3% |  |
|  | | | | | | 18 - ≤24 hours | | | | 506 | (11.5) | |  | |  | 18.4% |  |
|  | | | | | | >24 hours | | | | 138 | (3.1) | |  | |  | 7.2% |  |
| Ischemia time | | | | | | ≤12 hours | | | | 1185 | (39.1) | |  | |  | 9.1% | 0.010 |
| if donors age < 65 years | | | | | | 12 - ≤18 hours | | | | 1314 | (43.4) | |  | |  | 10.7% |  |
|  | | | | | | 18 - ≤24 hours | | | | 413 | (13.6) | |  | |  | 14.3% |  |
|  | | | | | | >24 hours | | | | 116 | (3.8) | |  | |  | 6.9% |  |
| Ischemia time | | | | | | ≤12 hours | | | | 897 | (65.8) | |  | |  | 18.7% | 0.001 |
| if donors age ≥ 65 years | | | | | | 12 - ≤18 hours | | | | 352 | (25.8) | |  | |  | 23.0% |  |
|  | | | | | | 18 - ≤24 hours | | | | 93 | (6.8) | |  | |  | 36.6% |  |
|  | | | | | | >24 hours | | | | 22 | (1.6) | |  | |  | 9.1% |  |
| Preservation solution | | | | | | HTK | | | | 3804 | (86.2) | |  | |  | 13.7% | 0.217 |
|  | | | | | | UW | | | | 597 | (13.5) | |  | |  | 13.9% |  |
|  | | | | | | other | | | | 10 | (0.2) | |  | |  | 30.0% |  |
| Perfusion quality at recovery | | | | | | good | | | | 4278 | (97.5) | |  | |  | 13.5% | 0.002 |
|  | | | | | | inferior | | | | 109 | (2.5) | |  | |  | 23.9% |  |
| Graft-quality at recovery | | | | | | good | | | | 3965 | (90.7) | |  | |  | 12.8% | <0.001 |
|  | | | | | | inferior | | | | 405 | (9.3) | |  | |  | 23.7% |  |
| Graft: kidney side | | | | | | right | | | | 2249 | (51.0) | |  | |  | 14.0% | 0.652 |
|  | | | | | | left | | | | 2162 | (49.0) | |  | |  | 13.5% |  |
| Graft: number of arteries | | | | | | one | | | | 3553 | (80.5) | |  | |  | 13.8% | 0.571 |
|  | | | | | | multiple | | | | 858 | (19.5) | |  | |  | 13.3% |  |
| Graft: number of veins | | | | | | one | | | | 4149 | (94.1) | |  | |  | 13.8% | 0.906 |
|  | | | | | | multiple | | | | 262 | (5.9) | |  | |  | 13.4% |  |
| Graft: length of ureter | | | | | | long | | | | 4354 | (98.7) | |  | |  | 13.8% | 0.216 |
|  | | | | | | short | | | | 57 | (1.3) | |  | |  | 8.8% |  |
| Rescue allocation | | | | | | no | | | | 4170 | (94.5) | |  | |  | 13.7% | 0.620 |
| (see guideline [20-21]) | | | | | | yes | | | | 241 | (5.5) | |  | |  | 14.5% |  |
| Kidney-sharing | | | | | | local | | | | 919 | (20.8) | |  | |  | 14.9% | 0.355 |
|  | | | | | | national / regional | | | | 3492 | (79.2) | |  | |  | 13.4% |  |
| Gender match | | | D male, R male | | | | | | | 1477 | (33.5) | |  | |  | 13.4% | 0.081 |
| (D=donor, R=recipient) | | | D female, R male | | | | | | | 1300 | (29.5) | |  | |  | 15.4% |  |
|  | | | D male, R female | | | | | | | 849 | (19.2) | |  | |  | 13.8% |  |
|  | | | D female, R female | | | | | | | 785 | (17.8) | |  | |  | 11.6% |  |
| **Medication (at ET-report)** | | | | | | |  | | |  |  | |  | |  |  |  |
| Plasmaexpander | | no | | | | | | | | 3170 | (71.9) | |  | |  | 13.8% | 0.376 |
| (any since admission) | | yes | | | | | | | | 1241 | (28.1) | |  | |  | 13.6% |  |
| Norepinephrine | | none | | | | | | | | 1270 | (28.8) | |  | |  | 16.1% | 0.046 |
| (actual doses) | | ≤0.1 μg/kg/min | | | | | | | | 1604 | (36.4) | |  | |  | 13.8% |  |
|  | | ≤0.2 μg/kg/min | | | | | | | | 737 | (16.7) | |  | |  | 11.5% |  |
|  | | >0.2 μg/kg/min | | | | | | | | 800 | (18.1) | |  | |  | 11.9% |  |
| Norepinephrine | | none | | | | | | | | 894 | (20.3) | |  | |  | 16.3% | 0.029 |
| within last 24 hours | | ≤0.1 μg/kg/min | | | | | | | | 1788 | (40.5) | |  | |  | 14.3% |  |
|  | | ≤0.2 μg/kg/min | | | | | | | | 809 | (18.3) | |  | |  | 12.4% |  |
|  | | >0.2 μg/kg/min | | | | | | | | 920 | (20.9) | |  | |  | 11.4% |  |
| Dopamine or dobutamine | | | | | | | | no | | 3987 | (93.4) | |  | |  | 13.4% | 0.353 |
| actual or within last 24 hours | | | | | | | | yes | | 424 | (9.6) | |  | |  | 16.5% |  |
| Catecholamines | | no | | | | | | | | 1138 | (25.8) | |  | |  | 16.2% | 0.016 |
| (actual) | | yes | | | | | | | | 3273 | (74.2) | |  | |  | 12.9% |  |
| Catecholamines | | | | | | | | no | | 765 | (17.3) | |  | |  | 16.2% | 0.035 |
| within last 24 hours | | | | | | | | yes | | 3646 | (82.7) | |  | |  | 13.2% |  |
| Steroids | | no | | | | | | | | 3036 | (68.8) | |  | |  | 14.6% | 0.056 |
| (actual) | | yes | | | | | | | | 1375 | (31.2) | |  | |  | 11.9% |  |
| Steroids | | no | | | | | | | | 2885 | (65.4) | |  | |  | 14.6% | 0.083 |
| within last 24 hours | | yes | | | | | | | | 1526 | (34.6) | |  | |  | 12.2% |  |
| Antidiuretics | | no | | | | | | | | 3491 | (79.1) | |  | |  | 14.2% | 0.091 |
| (actual) | | yes | | | | | | | | 920 | (20.9) | |  | |  | 12.2% |  |
| Antidiuretics | | no | | | | | | | | 2816 | (63.8) | |  | |  | 14.0% | 0.346 |
| within last 24 hours | | yes | | | | | | | | 1595 | (36.2) | |  | |  | 13.3% |  |
| Diuretics | | no | | | | | | | | 4088 | (92.7) | |  | |  | 13.4% | 0.018 |
| (actual) | | yes | | | | | | | | 323 | (7.3) | |  | |  | 17.6% |  |
| Diuretics within | | no | | | | | | | | 3921 | (89.9) | |  | |  | 13.4% | 0.046 |
| within last 24 hours | | yes | | | | | | | | 490 | (11.1) | |  | |  | 16.3% |  |
| Insulin | | no | | | | | | | | 3656 | (82.9) | |  | |  | 14.1% | 0.176 |
| (actual) | | yes | | | | | | | | 755 | (17.1) | |  | |  | 11.9% |  |
| Insulin | | no | | | | | | | | 3564 | (80.8) | |  | |  | 14.1% | 0.317 |
| within last 24 hours | | yes | | | | | | | | 847 | (19.2) | |  | |  | 12.4% |  |
| Antihypertonica | | no | | | | | | | | 3744 | (84.9) | |  | |  | 13.1% | 0.018 |
| (since admission) | | yes | | | | | | | | 667 | (15.1) | |  | |  | 17.1% |  |
| Therapeutic antibiotics | | no | | | | | | | | 2855 | (64.7) | |  | |  | 14.3% | 0.182 |
| (since admission) | | yes | | | | | | | | 1556 | (35.3) | |  | |  | 12.7% |  |
| **Additional Diagnosis** | |  | | | | | | | |  |  | |  | |  |  |  |
| Previous malignancy* | | not reported | | | | | | | | 4325 | (98.0) | |  | |  | 13.8% | 0.633 |
|  | | tumour | | | | | | | | 86 | (1.9) | |  | |  | 11.6% |  |
| History of arterial hypertension | | | | | | not reported | | | | 2568 | (58.2) | |  | |  | 12.3% | 0.001 |
|  | | | | | | reported | | | | 1843 | (41.8) | |  | |  | 15.7% |  |
| History of diabetes | | | | | not reported | | | | | 4240 | (96.1) | |  | |  | 13.6% | 0.097 |
|  | | | | | reported | | | | | 171 | (3.9) | |  | |  | 17.5% |  |
| History of arteriosclerosis | | | | | not reported | | | | | 4101 | (93.0) | |  | |  | 13.6% | 0.572 |
|  | | | | | reported | | | | | 310 | (7.0) | |  | |  | 15.5% |  |
| History of coronary heart disease | | | | | | | not reported | | | 3584 | (81.3) | |  | |  | 13.3% | 0.027 |
|  | | | | | reported | | | | | 827 | (18.7) | |  | |  | 15.7% |  |
| History of drug abuse* | | | | | not reported | | | | | 4338 | (98.3) | |  | |  | 13.8% | 0.221 |
|  | | | | | reported | | | | | 73 | (1.7) | |  | |  | 8.2% |  |
| History of smoking | | | | | not reported | | | | | 3177 | (72.0) | |  | |  | 14.3% | 0.181 |
|  | | | | | reported | | | | | 1234 | (28.0) | |  | |  | 12.4% |  |
| History of alcohol abuse | | | | | not reported | | | | | 3570 | (80.9) | |  | |  | 13.8% | 0.709 |
|  | | | | | reported | | | | | 841 | (19.1) | |  | |  | 13.3% |  |
| Hepatitis B* | anti HBc- / HBsAg- | | | | | | | | | 4173 | (94.6) | |  | |  | 13.7% | 0.616 |
|  | anti-HBc+ or HBsAg+ | | | | | | | | | 238 | (5.4) | |  | |  | 14.7% |  |
| Hepatitis C* | | | | | anti-HCV- | | | | | 4381 | (99.3) | |  | |  | 13.7% | 0.961 |
|  | | | | | anti-HCV + | | | | | 30 | (0.7) | |  | |  | 13.3% |  |
| Contact to cytomegaly virus | | | | | anti-CMV- | | | | | 1723 | (39.1) | |  | |  | 12.0% | 0.022 |
|  | | | | | anti-CMV+ | | | | | 2688 | (60.9) | |  | |  | 14.8% |  |
| Acute thoracic trauma | | | | | not reported | | | | | 4159 | (94.3) | |  | |  | 13.8% | 0.337 |
|  | | | | | reported | | | | | 252 | (5.7) | |  | |  | 11.9% |  |
| Acute sepsis* | | | | | not reported | | | | | 4379 | (99.3) | |  | |  | 13.8% | 0.529 |
|  | | | | | acute recovery | | | | | 32 | (0.7) | |  | |  | 9.4% |  |
| Acute meningitis* | | | | | not reported | | | | | 4372 | (99.1) | |  | |  | 13.8% | 0.170 |
|  | | | | | acute recovery | | | | | 39 | (0.9) | |  | |  | 5.1% |  |
| Pancreatitis | | | | | not reported | | | | | 4315 | (97.8) | |  | |  | 13.8% | 0.519 |
|  | | | | | reported | | | | | 96 | (2.2) | |  | |  | 11.5% |  |
| Acute pneumonia | | | | | not reported | | | | | 2964 | (67.2) | |  | |  | 14.0% | 0.364 |
|  | | | | | reported | | | | | 1447 | (32.8) | |  | |  | 13.3% |  |
| Left ventricular hypertrophy in | | | | | | | | none | | 1686 | (76.1) | |  | |  | 10.7% | 0.744 |
| echocardiography# | | | | | | | | moderate | | 373 | (16.8) | |  | |  | 10.7% |  |
|  | | | | | | | | severe | | 157 | (7.1) | |  | |  | 12.7% |  |
| **Laboratory data** | | | |  | | | | | |  |  |  | |  | |  |  |
| CK at admission | | | | lU/l IFCC | | | | | | 3723 |  | 168 | | 83-401 | | 1.000 [1.000-1.000] | 0.198 |
| CK lowest value | | | | lU/l IFCC | | | | | | 3723 |  | 134 | | 65-328 | | 1.000 [1.000-1.000] | 0.142 |
| CK peak value | | | | lU/l IFCC | | | | | | 3721 |  | 244 | | 102-630 | | 1.000 [1.000-1.000] | 0.222 |
| CK at ET report | | | | lU/l IFCC | | | | | | 3723 |  | 188 | | 76-525 | | 1.000 [1.000-1.000] | 0.163 |
| CK-MB at ET report | | | | lU/l IFCC | | | | | | 2253 |  | 27 | | 17-54 | | 0.999 [0.998-1.001] | 0.280 |
| AST at admission | | | | lU/l IFCC | | | | | | 4314 |  | 43 | | 27-82 | | 1.000 [0.999-1.000] | 0.169 |
| AST lowest value | | | | lU/l IFCC | | | | | | 4314 |  | 37 | | 25-66 | | 1.000 [0.999-1.000] | 0.667 |
| AST peak value | | | | lU/l IFCC | | | | | | 4314 |  | 57 | | 34-114 | | 1.000 [1.000-1.000] | 0.191 |
| AST at ET report | | | | lU/l IFCC | | | | | | 4314 |  | 48 | | 30-87 | | 1.000 [0.999-1.000] | 0.207 |
| ALT at admission | | | | lU/l IFCC | | | | | | 4325 |  | 31 | | 19-59 | | 1.000 [0.999-1.000] | 0.108 |
| ALT lowest value | | | | lU/l IFCC | | | | | | 4325 |  | 26 | | 17-49 | | 1.000 [0.999-1.000] | 0.466 |
| ALT peak value | | | | lU/l IFCC | | | | | | 4325 |  | 38 | | 22-79 | | 1.000 [0.999-1.000] | 0.035 |
| ALT at ET report | | | | lU/l IFCC | | | | | | 4325 |  | 32 | | 20-63 | | 0.999 [0.999-1.000] | 0.056 |
| yGT at admission | | | | lU/l IFCC | | | | | | 4240 |  | 40 | | 21-97 | | 1.000 [1.000-1.000] | 0.940 |
| yGT lowest value | | | | lU/l IFCC | | | | | | 4240 |  | 36 | | 19-86 | | 1.000 [0.999-1.001] | 0.984 |
| yGT peak value | | | | lU/l IFCC | | | | | | 4244 |  | 56 | | 24-135 | | 1.000 [1.000-1.000] | 0.980 |
| yGT at ET report | | | | lU/l IFCC | | | | | | 4244 |  | 49 | | 22-118 | | 1.000 [0.999-1.000] | 0.898 |
| AP at ET report | | | | lU/l IFCC | | | | | | 3793 |  | 74 | | 55-106 | | 1.000 [0.999-1.000] | 0.802 |
| LDH at ET report | | | | lU/l IFCC | | | | | | 3720 |  | 285 | | 212-420 | | 1.000 [0.999-1.000] | 0.292 |
| Sodium at admission | | | | mmol/l | | | | | | 4379 |  | 141 | | 138-148 | | 0.992 [0.982-1.002] | 0.131 |
| Sodium lowest value | | | | mmol/l | | | | | | 4370 |  | 140 | | 137-146 | | 0.994 [0.983-1.005] | 0.261 |
| Sodium peak value | | | | mmol/l | | | | | | 4388 |  | 150 | | 144-156 | | 0.988 [0.980-0.997] | 0.008 |
| Sodium at ET report | | | | mmol/l | | | | | | 4384 |  | 148 | | 142-153 | | 0.992 [0.983-1.001] | 0.088 |
| Glucose at admission | | | | mmol/l | | | | | | 4140 |  | 8.0 | | 6.7-10.2 | | 1.006 [0.985-1.027] | 0.576 |
| Glucose lowest value | | | | mmol/l | | | | | | 4136 |  | 7.0 | | 6.0-8.7 | | 1.016 [0.989-1.043] | 0.241 |
| Glucose peak value | | | | mmol/l | | | | | | 4140 |  | 8.9 | | 7.0-11.5 | | 1.008 [0.989-1.028] | 0.390 |
| Glucose at ET report | | | | mmol/l | | | | | | 4138 |  | 7.9 | | 6.4-10.0 | | 1.016 [0.994-1.038] | 0.152 |
| Creatinine at admission | | | | μmol/l | | | | | | 4400 |  | 76.9 | | 61.9-97.2 | | 1.001 [1.000-1.003] | 0.104 |
| Creatinine lowest value | | | | μmol/l | | | | | | 4401 |  | 70.7 | | 53.1-88.4 | | 1.001 [1.000-1.003] | 0.094 |
| Creatinine peak value | | | | μmol/l | | | | | | 4398 |  | 88.4 | | 68.1-118.5 | | 1.001 [1.000-1.001] | 0.298 |
| Creatinine at ET report | | | | μmol/l | | | | | | 4399 |  | 79.6 | | 61.9-110 | | 1.000 [0.999-1.002] | 0.389 |
| Bilirubin at admission | | | | μmol/l | | | | | | 4174 |  | 10.3 | | 6.8-17.1 | | 1.001 [0.997-1.004] | 0.708 |
| Bilirubin lowest value | | | | μmol/l | | | | | | 4176 |  | 10.0 | | 6.8-15.4 | | 1.001 [0.998-1.005] | 0.371 |
| Bilirubin peak value | | | | μmol/l | | | | | | 4174 |  | 12.1 | | 8.6-19.7 | | 1.000 [0.997-1.003] | 0.929 |
| Bilirubin at ET report | | | | μmol/l | | | | | | 4176 |  | 11.2 | | 7.0-18.1 | | 1.001 [0.998-1.004] | 0.397 |
| Haemoglobin at admission | | | | g/dl | | | | | | 4369 |  | 12.2 | | 10.4-14.0 | | 1.020 [0.987-1.055] | 0.230 |
| Haemoglobin lowest value | | | | g/dl | | | | | | 4367 |  | 10.5 | | 9.0-12.0 | | 1.025 [0.987-1.064] | 0.195 |
| Haemoglobin peak value | | | | g/dl | | | | | | 4372 |  | 12.4 | | 10.6-14.1 | | 1.021 [0.986-1.057] | 0.238 |
| Haemoglobin at ET report | | | | g/dl | | | | | | 4377 |  | 10.6 | | 9.3-12.2 | | 1.026 [0.987-1.067] | 0.189 |
| Leucocytes at admission | | | | G/l | | | | | | 4394 |  | 12.3 | | 9.4-16.3 | | 0.990 [0.977-1.004] | 0.170 |
| Leucocytes lowest value | | | | G/l | | | | | | 4393 |  | 10.9 | | 8.5-14.1 | | 0.994 [0.979-1.010] | 0.481 |
| Leucocytes peak value | | | | G/l | | | | | | 4394 |  | 14.6 | | 11.2-19.2 | | 0.995 [0.983-1.006] | 0.365 |
| Leucocytes at ET report | | | | G/l | | | | | | 4393 |  | 12.8 | | 9.9-17.1 | | 0.999 [0.986-1.012] | 0.890 |
| Thrombocytes at ET report | | | | T/l | | | | | | 4360 |  | 175.0 | | 123.0-238.0 | | 1.000 [0.999-1.001] | 0.610 |
| CRP at admission | | | | mg/l | | | | | | 3693 |  | 38.0 | | 5.0-144.0 | | 0.999 [0.998-1.000] | 0.093 |
| CRP lowest value | | | | mg/ | | | | | | 3691 |  | 33.0 | | 5.0-137.3 | | 0.999 [0.998-1.000] | 0.111 |
| CRP peak value | | | | mg/ | | | | | | 3981 |  | 145.0 | | 68.8-227.0 | | 0.999 [0.999-1.000] | 0.040 |
| CRP at ET report | | | | mg/ | | | | | | 3981 |  | 136.6 | | 61.0-219.0 | | 0.999 [0.999-1.000] | 0.051 |
| paO_2_/FIO_2_ at admission | | | | mmHg | | | | | | 3251 |  | 275.0 | | 199.0-370.0 | | 0.999 [0.998-1.000] | 0.053 |
| paO_2_/FIO_2_ lowest value | | | | mmHg | | | | | | 3250 |  | 260.0 | | 189.0-350.0 | | 1.000 [0.999-1.000] | 0.297 |
| paO_2_/FIO_2_ peak value | | | | mmHg | | | | | | 3249 |  | 298.0 | | 210.5-397.0 | | 0.999 [0.998-1.000] | 0.030 |
| paO_2_/FIO_2_ at ET report | | | | mmHg | | | | | | 3250 |  | 280.0 | | 200.0-375.0 | | 0.999 [0.999-1.000] | 0.179 |
| Urine-stix: protein | | | | none or (+) | | | | | | 3957 | (99.6) |  | |  | | 13.8% | 0.894 |
|  | | | | reactive | | | | | | 16 | (0.4) |  | |  | | 12.5% |  |
| Urine-stix: haemoglobin** | | | | none or (+) | | | | | | 1604 | (92.1) |  | |  | | 14.2% | 0.010 |
|  | | | | reactive | | | | | | 138 | (7.9) |  | |  | | 5.8% |  |
| Urine-stix: leucocytes | | | | none or (+) | | | | | | 2503 | (98.0) |  | |  | | 13.8% | 0.655 |
|  | | | | reactive | | | | | | 52 | (2.0) |  | |  | | 15.4% |  |
| Urine-stix: bacteria | | | | none or (+) | | | | | | 1262 | (94.0) |  | |  | | 13.9% | 0.859 |
|  | | | | reactive | | | | | | 80 | (6.0) |  | |  | | 13.8% |  |
|  | | | |  | | | | | |  |  |  | |  | |  |  |

*One of these diagnoses classifies an expanded criteria donor according to the German Medical Association [32-33].

#Only performed for potential heart donors. Therefore many missing values.

**Table e2:** Recipient characteristics used in univariate analyses of graft-survival after isolated kidney transplantation (KTX). For interval-scaled parameters absolute numbers, median, interquartile range, risk ratio (with 95% confidence interval) and p-values (Cox regression) are shown. For nominal and categorical parameters absolute numbers, proportions, the percentage of graft-failures and p-values (log-rank test) are shown.

| **Basic recipient data** | | | **Unit of analysis or factor level** | | **n** | **(%)** | **Median** | **Interquartile range** | **Graft-failure (%) or Hazard Ratio [95%-CI]** | **p-value** |  |
| --- | --- | --- | --- | --- | --- | --- | --- | --- | --- | --- | --- |
| Age | | | year | | 4411 |  | 56 | 46-65 | 1.028 [1.021-1.035] | <0.001 |  |
| Weight | | | kg | | 4411 |  | 75.0 | 65.0-86.0 | 1.003 [1.000-1.006] | 0.057 |  |
| Size | | | cm | | 4411 |  | 172.0 | 165.0-178.0 | 0.996 [0.991-1.002] | 0.164 |  |
| Time on dialysis before KTX | | | days | | 4346 |  | 2251 | 1239-2823 | 1.000 [1.000-1.000] | 0.141 |  |
| Gender | | | female | | 1634 | (37.0) |  |  | 12.7% | 0.145 | |
|  | | | male | | 2777 | (63.0) |  |  | 14.3% |  |  |
| **Comorbidities before KTX** | | |  | |  |  |  |  |  |  | |
| Arterial hypertension | | | not reported | | 688 | (15.6) |  |  | 13.4% | 0.811 | |
|  | | | reported | | 3723 | (84.4) |  |  | 13.8% |  |  |
| Diabetes | | | not reported | | 4035 | (91.5) |  |  | 13.4% | <0.001 | |
|  | | | reported | | 376 | (8.5) |  |  | 17.0% |  |  |
| Coronary heart disease | | | not reported | | 3351 | (76.0) |  |  | 11.9% | <0.001 | |
|  | | | reported | | 1060 | (24.0) |  |  | 19.4% |  |  |
| Peripheral artery occlusion | | | not reported | | 3908 | (88.6) |  |  | 12.8% | <0.001 | |
| disease | | | reported | | 503 | (11.4) |  |  | 21.1% |  |  |
| Any cardiovascular disease | | | not reported | | 2932 | (66.5) |  |  | 11.3% | <0.001 |  |
| (summary of above) | | | reported | | 1479 | (33.5) |  |  | 18.6% |  | |
| Cerebrovascular insult | | | not reported | | 4267 | (96.7) |  |  | 13.6% | 0.207 |  |
|  | | | reported | | 144 | (3.3) |  |  | 16.7% |  | |
| Chronic obstructive lung disease | | | not reported | | 4251 | (96.4) |  |  | 13.5% | 0.086 |  |
|  | | | reported | | 160 | (3.6) |  |  | 19.4% |  | |
| Chronic hepatitis | | | not reported | | 4246 | (96.3) |  |  | 13.7% | 0.398 | |
|  | | | reported | | 165 | (3.7) |  |  | 15.8% |  |  |
| Other co-morbidities | | | not reported | | 1649 | (37.4) |  |  | 13.3% | 0.468 |  |
|  | | | reported | | 2762 | (62.6) |  |  | 14.0% |  |  |
| **Immunological risks before KTX** | | | |  |  |  |  |  |  |  |  |
| Retransplantation | | | 1st KTX | | 3779 | (85,7) |  |  | 12.7% | <0.001 |  |
|  | | | 2nd KTX | | 525 | (11.9) |  |  | 19.4% |  |  |
|  | | | 3rd or more KTX | | 107 | (2.4) |  |  | 21.5% |  |  |
| Panel reactive antibody | | | % | | 4411 |  | 0.0 | 0.0-4.0 | 1.006 [1.003-1.009] | <0.001 |  |
| (peak value) | | | 0-5% | | 3511 | (79.6) |  |  | 13.2% | 0.020 |  |
|  | | | 6-84% | | 745 | (16.9) |  |  | 15.2% |  |  |
|  | | | 85-100% | | 155 | (3.5) |  |  | 20.0% |  |  |
| Panel reactive antibody | | | % | | 4411 |  | 0.0 | 0.0-0.0 | 1.008 [1.003-1.012] | <0.001 |  |
| (last value before KTX) | | | 0-5% | | 4019 | (91.1) |  |  | 13.3% | 0.007 |  |
|  | | | 6-84% | | 345 | (7.8) |  |  | 18.0% |  |  |
|  | | | 85-100% | | 47 | (1.1) |  |  | 23.4% |  |  |
| HLA-mismatch (A+B-+DR) | | | 0 | | 547 | (12.7) |  |  | 8.8% | <0.001 |  |
| (broad antigen) | | | 1 | | 322 | (7.3) |  |  | 12.7% |  |  |
|  | | | 2 | | 882 | (20.0) |  |  | 11.3% |  |  |
|  | | | 3 | | 1119 | (25.4) |  |  | 13.5% |  |  |
|  | | | 4 | | 764 | (6.8) |  |  | 14.8% |  |  |
|  | | | 5 | | 527 | (12.0) |  |  | 20.3% |  |  |
|  | | | 6 | | 248 | (5.6) |  |  | 18.1% |  |  |
| HLA-mismatch A* | | | 0 | | 1326 | (44.5) |  |  | 10.1% | 0.745 |  |
| (broad antigen) | | | 1 | | 1422 | (47.8) |  |  | 11.1% |  |  |
|  | | | 2 | | 226 | (7.5) |  |  | 11.1% |  |  |
| HLA-mismatch B* | | | 0 | | 908 | (30.5) |  |  | 8.6% | 0.040 |  |
| (broad antigen) | | | 1 | | 1578 | (53.1) |  |  | 11.2% |  |  |
|  | | | 2 | | 488 | (16.4) |  |  | 12.9% |  |  |
| HLA-mismatch DR* | | | 0 | | 1117 | (37.6) |  |  | 8.7% | 0.030 |  |
| (broad antigen) | | | 1 | | 1546 | (51.9) |  |  | 11.7% |  |  |
|  | | | 2 | | 311 | (10.5) |  |  | 12.5% |  |  |
| **Postoperative course of KTX** | | |  | |  |  |  |  |  |  |  |
| Postoperative stay in hospital | | | days | | 4411 |  | 21 | 16-29 | 1.018 [1.016-1.021] | <0.001 |  |
| Duration of KTX-operation | | | minutes | | 4411 |  | 160 | 129-198 | 1.002 [1.000-1.003] | 0.009 |  |
| Postoperative dialysis | | | number | | 4103 |  | 0 | 0-2 | 1.078 [1.062-1.095] | <0.001 |  |
| Rejections postoperative | | | 0 | | 3667 | (83.1) |  |  | 11.9% | <0.001 |  |
|  | | | 1 | | 635 | (14.4) |  |  | 20.3% |  |  |
|  | | | ≥2 | | 109 | (2.5) |  |  | 38.5% |  |  |
| Induction therapy at KTX | | | none | | 1772 | (40.2) |  |  | 15.0% | 0.162 |  |
|  | | | ATG, OKT3 or other | | 668 | (15.1) |  |  | 15.1% |  |  |
|  | | | Il2-receptor antibodies | | 1971 | (44.7) |  |  | 12.2% |  |  |
| Postoperative complications | | Not reported | | | 3630 | (82.2) |  |  | 11.3% | <0.001 |  |
| (worst case scenario in | 1. Urine leak or lymphocele | | | | 330 | (7.5) |  |  | 12.4% |  |  |
| case of multiple counts) | 2. Wound infection / dehiscence | | | | 386 | (8.8) |  |  | 25.9% |  |  |
|  | 3. Thrombosis (a. or v. renalis) | | | | 65 | (1.5) |  |  | 83.1% |  |  |

*Missing values because only donors younger than 65 years are considered (due to technical limitations within ESP).

**
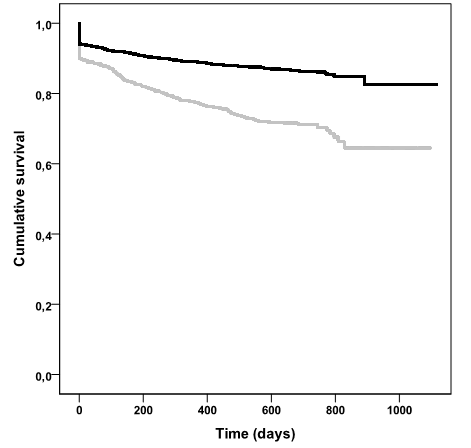
**

Supplementary Figure e1: Kaplan-Maier estimates of graft survival after kidney transplantation according to donor age (left: cases grouped into donor age < 65 years (n=3028; black solid line) and donor age ≥65 years (n=1364; grey solid line); p<0.001.

**
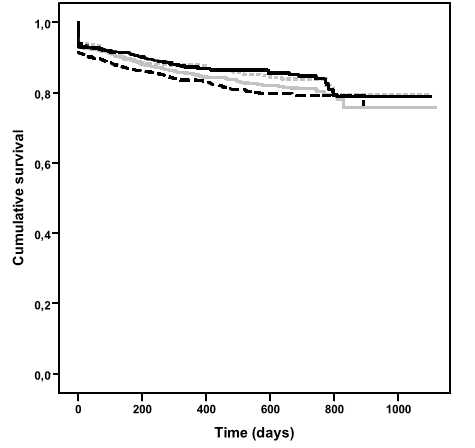
**

Supplementary Figure e2: Kaplan-Maier estimates of graft survival after kidney transplantation according to Norepinephrine dose before recovery: case grouped into none (n=894; black dashed line), ≤0.1µg/kg/min (n=1788; grey solid line); ≤0.2 µg/kg/min (n=809; grey dashed line); >0.2 µg/kg/min (n=920; black solid line); p=0.046)

**
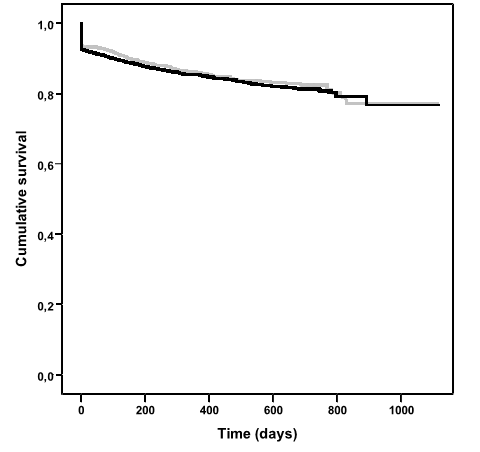
**

Supplementary Figure e3: Kaplan-Maier estimates of graft survival after kidney transplantation according to use of any colloids during donor maintenance: cases without colloids used (n=3170; black line) and colloids used (n=1241; grey line); p=0.376)
